# Assisted reproductive technology and risk of ovarian cancer and borderline tumors in parous women: a population-based cohort study

**DOI:** 10.1007/s10654-019-00540-3

**Published:** 2019-08-03

**Authors:** Frida E. Lundberg, Anna L. V. Johansson, Kenny Rodriguez-Wallberg, Kristina Gemzell-Danielsson, Anastasia N. Iliadou

**Affiliations:** 1 Department of Medical Epidemiology and Biostatistics, Karolinska Institutet, Stockholm, Sweden; 2 Department of Oncology-Pathology, Karolinska Institutet, Stockholm, Sweden; 3 Cancer Registry of Norway, Oslo, Norway; 4 Department of Gynecology and Reproduction, Section of Reproductive Medicine, Karolinska University Hospital, Stockholm, Sweden; 5 Division of Obstetrics and Gynecology, Department of Women’s and Children’s Health, Karolinska Institutet and Karolinska University Hospital, WHO Collaborating Centre, Stockholm, Sweden

**Keywords:** Infertility, Assisted reproductive technology, In vitro fertilization, Ovarian stimulation, Ovarian cancer, Borderline ovarian tumor

## Abstract

The study aimed to investigate if assisted reproductive technology (ART) treatment or a diagnosis of infertility were associated with the risk of ovarian cancer or borderline ovarian tumors (BOT) in parous women. In a population-based register study of 1,340,097 women with a first live birth in Sweden 1982–2012, the relationship between ART treatments, infertility and incidence of ovarian cancer or BOT were investigated using Cox regression analysis. In the cohort, 38,025 women gave birth following ART, 49,208 following an infertility diagnosis but no ART and 1,252,864 without infertility diagnosis or ART. During follow-up, 991 women were diagnosed with ovarian cancer and 747 with BOT. Women who gave birth following ART had higher incidence of both ovarian cancer (adjusted hazard ratio [aHR] 2.43, 95% confidence interval [CI] 1.73–3.42) and BOT (aHR 1.91, 95% CI 1.27–2.86), compared to women without infertility. Compared to women with infertility diagnoses and non-ART births, women with ART births also had a higher incidence of ovarian cancer (aHR 1.79, 95% CI 1.18–2.71) and BOT (aHR 1.48, 95% CI 0.90–2.44). Our results suggest that women who have gone through ART have a higher risk of ovarian cancer and BOT. At least part of that risk seems to be due to the underlying infertility and not the treatment per se, since the increased risk was smaller when comparing to other infertile women. As ART treatments are becoming more common and ovarian cancer usually occur in women of advanced age, larger studies with longer follow-up are needed in order to confirm or refute our findings.

**Electronic supplementary material:**

The online version of this article (10.1007/s10654-019-00540-3) contains supplementary material, which is available to authorized users.

## Introduction

Assisted reproductive technology (ART) treatments apply to the systemic use of high doses of gonadotropins to stimulate multiple ovarian follicle recruitment and the subsequent puncturing of the ovaries for aspiration of mature oocytes. As the treatments increase endogenous estrogen levels and ovarian cell proliferation, ART has been suspected to influence the risk of ovarian cancer [1].

Ovarian cancer is a rare and life-threatening disease, accounting for 3.6% of incident cancers and 4.3% of cancer-specific mortality among women worldwide [2]. Risk factors for ovarian cancer include nulliparity, early menarche, late menopause and ovarian cancer in first degree relatives [3], while increasing age at first birth is negatively associated with ovarian cancer risk [4, 5]. Some studies have also found an increased risk of ovarian cancer among women with endometriosis [6–8], irregular menstruation [9], and women evaluated for infertility [10, 11].

Several studies have investigated the risk for ovarian cancer in women who have gone through ART with inconclusive results. Most studies to date have compared the risk of ovarian cancer after ART to that of the general population [12–18]. While three of these studies reported an increased risk among women treated with ART [13, 16, 18], it is unclear whether these associations were due to the treatments or the infertility per se. In studies comparing ovarian cancer risk after ART to the risk in other infertile women, the results have been equally inconclusive [7, 19, 20].

Some previous studies have also found an increased risk of borderline ovarian tumors (BOT) following ART [12, 18, 20, 21]. These tumors are more common in younger women and have a better prognosis than ovarian cancer [22].

The objective of the present population-based cohort study was to investigate the associations between ART and the incidence of invasive ovarian cancer and borderline tumors, and the role of the underlying infertility for the studied associations.

## Materials and methods

### Study population

The study cohort included all women who had their first live birth between 1982 and 2012 recorded in the population-based Swedish Multi-Generation Register (MGR) (n = 1,535,678). The MGR links all parents and children born since 1932 and residing in Sweden 1961 or later [23]. We used the personal identification number assigned to all Swedish residents for linking the MGR to other Swedish registers.

Women with invalid personal identity numbers (n = 1876), not residing in Sweden at the start of follow-up (n = 189,110), diagnosed with any malignant disease before start of follow-up (n = 4481) or with bilateral oophorectomy before start of follow-up (n = 114) were excluded from the study population, leaving a cohort of 1,340,097 women. Only women with complete information on all covariates were included in the analyses (n = 1,315,924). In the analyses of BOT incidence, 183 women who were diagnosed with a BOT before start of follow-up were also excluded.

### Exposure information

Infertility, defined as trying to conceive for at least 1 year without success, is normally diagnosed in couples who seek medical help to have a child. Women diagnosed with infertility (International Classification of Diseases [ICD] version 7 code 636; ICD-8 628; ICD-9 628; ICD-10 N97) were identified using the National Patient Register, where in-patient care has been recorded since 1964 and specialist out-patient care since 2001 [24].

Information on ART cycles that resulted in a live birth was obtained from the Swedish National Board of Health and Welfare, which recorded information from all ART clinics in Sweden during the years 1982–2006. Since 2007 all ART cycles have been recorded in the National Quality Registry of Assisted Reproductive Technology (Q-IVF). Using the Q-IVF information, ART births following fresh and frozen embryo transfers from standard in vitro fertilization (IVF) and intracytoplasmic sperm injection (ICSI) were identified between 2007 and 2012. Ovarian stimulation for ART has been performed with gonadotropins alone in Sweden since the mid-1990s, before which clomiphene citrate (alone or in combination with gonadotropins) was also used [16].

### Ovarian cancer and borderline ovarian tumors (BOT)

Since 1958, diagnoses of cancer in all Swedish residents have been recorded in the Swedish Cancer Register (SCR). The register includes date of diagnosis, hospital, tumor site coded using the 7th and current version of ICD and morphology using the WHO/HS/CANC/24.1 histology codes [25]. Since 1993 histology is also coded using ICD-O/2 codes. The estimated completeness of the SCR for solid tumors is over 95% [26].

In the present study, ovarian cancers were defined as morphologically verified malignant tumors of the ovary, fallopian tube and broad ligaments (ICD-7 175), or peritoneum (ICD-7 158). For cases diagnosed 1993 or later, subtypes of ovarian cancer were defined according to ICD-O/2 codes as serous, mucinous, endometrioid, clear-cell, other or unspecified carcinoma, and non-epithelial tumors. Borderline ovarian tumors were defined according to ICD-7 175 and histology code 094/b. Women with a first diagnosis of ovarian cancer or BOT during follow-up were considered cases. Women with any diagnosis of malignant disease (ICD-7 140-205) before start of follow-up were excluded, and those with a malignant disease other than ovarian during the study period were censored at date of diagnosis. By combining information in the MGR with the SCR, family history of cancer was defined as having a biological mother or sister with breast or ovarian cancer.

### Covariates

The woman’s parity and birthdate of each child was obtained by combining information in the MGR and the Total Population Register. The Medical Birth Register (MBR) was used to obtain gestational length, smoking during the first trimester of pregnancy, height, pre-pregnancy weight and previous still births. Date of conception was calculated by subtracting the gestational length from the date of childbirth. Where gestational length was missing (142,598 births to 124,524 women), 280 days was used. Body mass index (BMI) was calculated from height and pre-pregnancy weight as kg/m^2^.

From the Total Population Register we obtained each woman’s date and country of birth as well as any migrations in or out of Sweden. Date of death was obtained from the Cause of Death Register and highest attained education level from the Education Register. The National Patient Register was used to identify women who had gone through bilateral oophorectomy, salpingectomy, and hysterectomy.

### Statistical analyses

Person-time at risk was accrued from the date of conception of the first live birth until the date of first ovarian cancer diagnosis, or censoring at date of other cancer diagnosis, death, emigration, start of pregnancy with fourth child, bilateral oophorectomy, 60th birthday or the end of follow-up in December 2012, whichever occurred first. The follow-up was restricted to before age 60, since no ART exposed woman had ovarian cancer or BOT after age 60. Similarly, to simplify the analysis, the follow-up was restricted to women with at most three children, since all but one case of ovarian cancer and one case of BOT occurred in women with less than four children. In the analysis of BOT incidence, follow-up time was also censored at date of ovarian cancer diagnosis. Women diagnosed with BOT and ovarian cancer on the same date were only counted as ovarian cancer cases (n = 5). Women diagnosed first with BOT and later with ovarian cancer were included as cases in both analyses (n = 4). Oophorectomy performed up to 60 days before diagnosis date of ovarian cancer (n = 42) or BOT (n = 36) was considered related to the tumor and not used for censoring in the respective analyses.

Cox proportional hazard models were used to estimate hazard ratios (HRs, which can be interpreted as incidence rate ratios), with 95% CIs for ovarian cancer and BOT, respectively, using attained age as the timescale. The main models compared incidence rates among women with ART birth and among women with infertility diagnoses but no ART to that of women with neither an infertility diagnosis nor ART. In order to assess confounding by the underlying infertility, HRs were also estimated using women with infertility diagnoses but no ART as the reference category.

Having an ART birth was entered into the models as a time-dependent exposure, i.e. women changed exposure category from the date of conception of an ART birth and were considered exposed to ART thereafter. Women who had a spontaneous (non-ART) birth prior to ART contributed person-time to first the unexposed and then the exposed group. Women with non-ART births were subdivided into two groups; with and without any diagnosis of infertility before end of follow-up.

Parity, calendar time (split into 10-year intervals), salpingectomy, and hysterectomy were included in the models as time-dependent covariates. Age at first birth, country of birth, education level, and family history of cancer were included as fixed covariates. The proportional hazards assumption was assessed using tests based on Schoenfeld residuals, and fulfilled for both outcomes BOT and ovarian cancer. There was no power to assess time-varying effects by recency of ART exposure.

To investigate associations with specific cancer subtypes, we performed separate analyses for each subtype of ovarian cancer while censoring at the other tumor types. In these analyses, follow-up started in January 1st 1993 or later.

For both outcomes, we performed five separate sensitivity analyses. First, we separated infertility diagnoses and ART treatments given before and after first birth to investigate potential differences between primary and secondary infertility. The interactions were assessed using likelihood ratio tests comparing these models to the main models. Secondly, we excluded cases and person-time during the first year of follow-up. Thirdly, we included BMI before the first pregnancy resulting in a live birth (index pregnancy) as a fixed covariate. Fourthly, we included smoking during index pregnancy in the same manner. For comparison, the age-adjusted and main multivariable adjusted models were also rerun in the subpopulations of women with information on BMI and smoking, respectively. Lastly, we excluded women born in non-Nordic countries since there may be different incidence rates of ovarian tumors in different populations.

Data was prepared using SAS software (version 9.4, SAS Institute Inc., Cary, N.C., USA) and statistical analyses performed using Stata (StataCorp. 2017. *Stata Statistical Software: Release 15.* College Station, TX: StataCorp LLC). All tests were two-sided with a significance level of 5%.

The Ethical Review Board in Stockholm, Sweden approved this study (ethical approval 2013/1849-31/2, amendment 2014/118-32).

## Results

Of the 1,340,097 parous women in the cohort, 991 women were diagnosed with ovarian cancer. More than 80% of the ovarian cancers were epithelial (n = 798). A small number of cancers were of peritoneal (n = 21), tubal (n = 31), and unspecified ovarian origin (n = 2). The mean age at ovarian cancer diagnosis was 44.2 years among women with ART birth, 44.8 years among women with infertility diagnoses and 42.5 years among women with no infertility diagnosis or ART birth. Among the 1,339,914 women with no BOT before follow-up start, 747 women were diagnosed with BOT. The mean age at BOT diagnosis was 41.6 years, 40.4 years and 39.8 years in the three exposure groups, respectively. The mean follow-up length was 9.5 years for women with ART birth, 13.2 years for women with infertility diagnoses and 14.6 years for women with no infertility diagnosis or ART birth. The crude incidence rate was 10.7 ovarian cancer cases per 100,000 person-years in women with ART birth, 8.7 in women with infertility diagnoses and 4.9 in women with no infertility diagnosis or ART birth. The corresponding rates of BOT were 8.8, 7.0 and 3.7 cases per 100,000 person-years, respectively.

### Population characteristics

Population characteristics are presented in Table 1. Nearly half of the women with ART births were born 1970–1979, while they were less likely to be born before 1960 and after 1980 compared to women with no ART birth. In the National Patient Register data available, 66.3% of women with ART birth and 3.8% of all women with no ART birth had at least one diagnosis of infertility. The majority of infertility diagnoses and ART treatments were given prior to the first childbirth. Compared to women with no ART births, women with ART births were more highly educated, while there was no difference in geographical origin or family history of cancer between the three groups. Women with infertility diagnoses and/or ART-births were older at their first birth and had fewer children at end of follow-up. Women with ART births were less likely to smoke during pregnancy and more likely to have higher BMI. BMI was also higher among women with infertility diagnoses and non-ART birth compared to women with no infertility diagnosis. Salpingectomy was more common among women with ART-births and women with infertility diagnoses were more likely to go through both salpingectomy and hysterectomy during follow-up. Women with ART-births and women with infertility diagnoses were also more likely to have had a still birth before their first live birth. The characteristics were similar when restricting to women with no BOT before follow-up start (Supplementary table 1).

**Table 1:** Characteristics of the study population by exposure to ART births and infertility

Characteristic	ART birth n = 38,025 (2.8%)	Infertility, no ART birth n = 49,208 (3.7%)	No infertility, no ART birth n = 1,252,864 (93.5%)
Birth year			
< 1960	2813 (7.4%)	9474 (19.3%)	172,604 (13.8%)
1960–1969	12,908 (33.9%)	14,038 (28.5%)	454,338 (36.3%)
1970–1979	18,716 (49.2%)	19,102 (38.8%)	412,459 (32.9%)
≥ 1980	3588 (9.4%)	6594 (13.4%)	213,463 (17.0%)
Diagnosis of infertility, ever	25,208 (66.3%)	49,208 (100%)	0
Age at infertility diagnosis, mean (± SD)	31.2 ± 4.2	30.8 ± 5.1	No diagnosis
ART/infertility diagnosis before first birth	32,458 (85.4%)	32,549 (66.1%)	No ART/diagnosis
Highest education achieved			
Compulsory school	1763 (4.6%)	4559 (9.3%)	107,913 (8.6%)
Secondary school	14,199 (37.3%)	21,642 (44.0%)	566,379 (45.2%)
Higher education	21,947 (57.7%)	22,722 (46.2%)	554,800 (44.3%)
Missing	116 (0.3%)	285 (0.6%)	23,772 (1.9%)
Country of birth			
Nordic country	33,165 (87.2%)	41,849 (85.0%)	1,107,301 (88.4%)
Non-nordic country	4860 (12.8%)	7359 (15.0%)	145,487 (11.6%)
Missing	0	0	76 (0.0%)
Family history of breast or ovarian cancer	2368 (6.2%)	2613 (5.3%)	69,205 (5.5%)
Parity at end of follow-up			
One child	14,729 (38.7%)	19,312 (39.2%)	299,259 (23.9%)
Two children	17,564 (46.2%)	21,445 (43.6%)	641,178 (51.2%)
Three children	5732 (15.1%)	8451 (17.2%)	312,427 (24.9%)
Age at first birth			
< 25 years	2229 (5.9%)	8933 (18.2%)	419,537 (33.5%)
25–29 years	7498 (19.7%)	14,852 (30.2%)	475,689 (38.0%)
30–34 years	15,595 (41.0%)	15,639 (31.8%)	271,788 (21.7%)
≥ 35 years	12,703 (33.4%)	9784 (19.9%)	85,850 (6.9%)
Bilateral oophorectomy	217 (0.6%)	350 (0.7%)	3633 (0.3%)
Salpingectomy	3100 (8.2%)	2669 (5.4%)	20,965 (1.7%)
Hysterectomy	547 (1.4%)	1139 (2.3%)	20,847 (1.7%)
BMI before index pregnancy			
< 18.5 kg/m^2^	678 (1.8%)	1430 (2.9%)	41,880 (3.3%)
18.5–24.9 kg/m^2^	20,873 (54.9%)	24,597 (50.0%)	652,539 (52.1%)
25.0–29.9 kg/m^2^	7690 (20.2%)	8337 (16.9%)	170,309 (13.6%)
≥ 30.0 kg/m^2^	2780 (7.3%)	4045 (8.2%)	61,074 (4.9%)
Missing	6004 (15.8%)	10,799 (21.9%)	327,062 (26.1%)
Smoking during index pregnancy			
No	33,039 (86.9%)	38,079 (77.4%)	934,843 (74.6%)
Yes	2065 (5.4%)	6685 (13.6%)	187,382 (15.0%)
Missing	2921 (7.7%)	4444 (9.0%)	130,639 (10.4%)
Stillbirth before index pregnancy			
No	37,190 (97.8%)	47,364 (96.3%)	1,200,424 (95.8%)
Yes	217 (0.6%)	356 (0.7%)	4955 (0.4%)
Missing	618 (1.6%)	1488 (3.0%)	47,485 (3.8%)

*ART* assisted reproductive technology, *BMI* body mass index

Data presented as number (%) unless otherwise specified

### Association between ART and incidence of ovarian cancer

In the age-adjusted analysis, the incidence of ovarian cancer was increased in women with infertility diagnoses (HR 1.46, 95% CI 1.11–1.91) and in women with ART births (HR 2.17, 95% CI 1.57–2.99) compared to women with no infertility diagnosis (Table 2). After adjusting for potential confounders, the risk was slightly attenuated in women with infertility diagnoses (aHR 1.36, 95% CI 1.03–1.79) but higher in women with ART births (aHR 2.43, 95% CI 1.73–3.42). When women with infertility diagnoses and non-ART birth were the reference group, the incidence of ovarian cancer was higher among women with ART births in the multivariable analysis (aHR 1.79, 95% CI 1.18–2.71). In stratified analyses (Supplementary table 2), primary infertility seemed to be associated with a higher incidence of ovarian cancer (aHR 1.46, 95% CI 1.07–2.01) than secondary infertility (aHR 1.13, 95% CI 0.67–1.92), although the confidence intervals were overlapping. Ovarian cancer incidence was similar in women with ART for first birth (aHR 2.45, 95% CI 1.71–3.51) and for subsequent birth only (aHR 2.38, 95% CI 0.89–6.39).

**Table 2:** Associations between infertility, ART and ovarian cancer incidence

	Cancer cases	Person-years	Age-adjusted	Multivariable^a^
			HR (95% CI)	HR (95% CI)
Population reference				
No infertility	894	18,211,037	1.00 (reference)	1.00 (reference)
Infertility (no ART)	56	670,335	1.46 (1.11–1.91)	1.36 (1.03–1.79)
ART birth	39	320,242	2.17 (1.57–2.99)	2.43 (1.73–3.42)
Infertile reference				
No infertility	894	18,211,037	0.68 (0.52–0.90)	0.73 (0.56–0.97)
Infertility (no ART)	56	670,335	1.00 (reference)	1.00 (reference)
ART birth	39	320,242	1.48 (0.98–2.23)	1.79 (1.18–2.71)

*ART* assisted reproductive technology, *CI* confidence interval, *HR* hazard ratio

^a^Adjusted for age, calendar time, parity, age at first birth, education level, country of birth, family history of breast or ovarian cancer, salpingectomy, and hysterectomy

Analyses separated by cancer subtype are presented in Supplementary table 3, among the women followed from 1993 (n = 1,318,439, 98.4% of the cohort). Compared to women with no infertility, the incidence of serous (aHR 1.66, 95% CI 1.11–2.47) and clear-cell tumors (aHR 2.97, 95% CI 1.03–8.59) was higher in women with infertility diagnoses in the multivariable adjusted analyses. Having had an ART birth was associated with a higher incidence of serous (aHR 2.08, 1.18–3.65), endometrioid (aHR 3.50, 95% CI 1.42–8.58), clear-cell (aHR 8.04, 95% CI 2.20–29.35) and other or unspecified carcinomas (aHR 3.98, 95% CI 1.71–9.28). Further, the aHR of mucinous tumors was 1.35 (95% CI 0.47–3.85) and of non-epithelial tumors 1.95 (95% CI 0.77–4.96) among women with ART births, compared to women with no infertility although the confidence intervals were wide.

After excluding the first year of follow-up (Supplementary table 4) the aHR of ovarian cancer among women with ART birth was 2.46 (95% CI 1.73–3.49) compared to women with no infertility diagnosis or ART birth, and 1.81 (95% CI 1.18–2.78) compared to women with infertility diagnoses and no ART birth. Adjusting for BMI (Supplementary table 5) or smoking during pregnancy (Supplementary table 6) did not substantially change the results from the main analyses, and neither did excluding women born in non-Nordic countries (Supplementary table 7).

### Association between ART and incidence of BOT

BOT incidence was higher in women with infertility diagnoses (HR 1.43, 95% CI 1.04–1.98) and women with ART births (HR 1.97, 95% CI 1.34–2.89) compared to women with no infertility diagnosis or ART birth in the age-adjusted analysis (Table 3). In the multivariable analysis, BOT incidence was higher among women with ART birth (aHR 1.91, 95% CI 1.27–2.86) and in women with infertility diagnoses (aHR 1.29, 95% CI 0.93–1.79). Compared to women with infertility diagnoses, the aHR of BOT was 1.48 (95% CI 0.90–2.44) in women with ART birth. When stratifying infertility or ART by first birth (Supplementary table 8), the incidence of BOT was higher in women with primary infertility (aHR 1.73, 95% CI 1.21–2.47), but not secondary infertility (aHR 0.55, 95% CI 0.25–1.23). Having ART for first birth was associated with higher BOT incidence (aHR 2.17, 95% CI 1.44–3.26), while there were no cases of BOT among women who had ART for subsequent birth only.

**Table 3:** Associations between infertility, ART and BOT incidence

	BOT cases	Person-years	Age-adjusted	Multivariable^a^
			HR (95% CI)	HR (95% CI)
Population reference				
No infertility	681	18,206,107	1.00 (reference)	1.00 (reference)
Infertility (no ART)	39	669,910	1.43 (1.04–1.98)	1.29 (0.93–1.79)
ART birth	27	319,978	1.97 (1.34–2.89)	1.91 (1.27–2.86)
Infertile reference				
No infertility	681	18,206,107	0.70 (0.51–0.96)	0.78 (0.56–1.08)
Infertility (no ART)	39	669,910	1.00 (reference)	1.00 (reference)
ART birth	27	319,978	1.37 (0.84–2.25)	1.48 (0.90–2.44)

*ART* assisted reproductive technology, *BOT* borderline ovarian tumor, *CI* confidence interval, *HR* hazard ratio

^a^Adjusted for age, calendar time, parity, age at first birth, education level, country of birth, family history of breast or ovarian cancer, salpingectomy, and hysterectomy

When excluding the first year of follow-up (Supplementary table 9), women with ART birth had an aHR of 2.03 for BOT (95% CI 1.34–3.07) compared to women with no infertility diagnosis, and 1.52 (95% CI 0.92–2.53) compared to women with infertility diagnoses. The main results did not change markedly after adjusting for either BMI (Supplementary Table 10) or smoking (Supplementary table 11), or when excluding women born in non-Nordic countries (Supplementary table 12).

## Discussion

Our results indicate that women who have gone through ART have an elevated risk of ovarian cancer and BOT. At least part of this effect seems to be associated with the underlying infertility, since the effect was smaller when compared to women with infertility diagnoses and non-ART birth. As both the causes and severity of infertility may differ between infertile women who conceived with and without ART, we cannot conclude if ART treatment itself may play a role in the development of ovarian cancer or BOT. The findings from analyses stratified by ovarian cancer subtype did not suggest that the risk is specifically higher for any histological subtype. Women who conceived using ART had a higher risk of serous, endometrioid, clear-cell and other or unspecified ovarian carcinoma, compared to women with no infertility or ART birth. However, these results are based on small numbers of cases and should be interpreted cautiously.

The strengths of our study include the large population and the practically complete follow-up of the women. By utilizing Swedish population-based registers we were able to accurately identify incident ovarian tumors as well as control for many important confounding variables while eliminating the risk of recall bias. In addition, the mean age at diagnosis was similar among women with and without ART births indicating that there was no detection bias due to closer surveillance of women going through ART. Some limitations need to be considered when interpreting our findings. Despite the large study population, our results were still based on few cases since the outcomes are rare and should be interpreted with caution, especially for ovarian cancer subtypes. The median age at diagnosis in Sweden is 62 years for ovarian cancer and 55 years for BOT [22]. The identified cases therefore consisted mainly of women with early onset disease. Further, there was no information available on the number of ART cycles each woman had gone through, the total gonadotropin dose or ART cycles that did not lead to live births. Therefore we were not able to investigate a dose–response relationship between ART use and ovarian cancer risk. Neither did we have information on oral contraceptive use. Oral contraceptive use has been associated with a lower risk of ovarian cancer [27], and could potentially bias the results if women who have gone through ART use less oral contraceptives or none at all after detection of their infertility. As the Swedish Patient Register does not cover primary health care, and specialist out-patient care has only been included since 2001, some women with infertility were likely classified as non-infertile. This could bias the results towards the null when comparing women with and without infertility diagnoses. It is also possible that the causes and severity of infertility differed between infertile women who conceived with and without ART. Additionally, the registry provided no information on whether the infertility was due to male or female factors, which could have further separated potential effects of treatment from that of the underlying infertility. Finally, we were not able to distinguish between high and low-grade serous carcinoma, or subtypes of borderline ovarian tumors.

A recent study from Great Britain found a higher risk of both invasive and borderline ovarian tumors among women who had assisted reproduction, compared to the general population [18]. The increased risk appeared limited to women with endometriosis or low parity. Since the cause of infertility was unavailable for most women in this study, we could not investigate the influence of endometriosis. A previous Swedish cohort study investigating cancer risk among women who gave birth after ART showed a higher risk of ovarian cancer compared to women with spontaneous conception [16], and a cohort study from Israel [13] also showed an increased risk of ovarian cancer associated with IVF treatments, although based on very few cases. Both of these last studies also used population control groups. However, most previous studies of cancer risk following ART have not found any statistically significant associations with ovarian cancer [7, 12, 14, 15, 17, 19, 20], including those comparing ART exposed women to untreated infertile women [7, 19, 20]. The 2016 guidelines published by the American Society for Reproductive Medicine (ASRM) concluded that “based on available data, there does not appear to be a meaningful increased risk of invasive ovarian cancer […] following the use of fertility drugs” [1]. However, several of the more recent studies have reported point estimates of between 1.3 and 1.6 for ovarian cancer after ART treatment [7, 12, 14, 19, 20]. These are also similar to the standardized incidence ratio of 1.4 reported in the British study [18], suggesting that the studies included in the ASRM review could have been underpowered to identify a modest increase in risk. In addition, while the Dutch study [20] did not find an overall association between ART and ovarian cancer, they reported an increased risk after ART among women with at least 15 years of follow-up. Due to age truncation in our study cohort, the majority of women with ART births were below 60 years of age at end of follow-up and no ovarian cancer was diagnosed in women above 60 years of age in this group. Since ovarian cancer is more likely to be diagnosed in women above 60 years of age, larger studies with longer follow-up are required to fully investigate the potential associations with infertility and ART. Similar to a recent Norwegian study [12], we found an increased risk of BOT in women with ART births compared to women who conceived spontaneously. Two previous studies have reported an increased risk of BOT also when comparing to other infertile women [20, 21]. In our study, the effect was smaller when comparing to women with infertility diagnoses, suggesting that the increased risk was confounded by the underlying infertility. The discrepancies in the results between studies could be due to differences between the study populations, for instance population size, number of ART exposed women and length of follow-up. There may also be socioeconomic differences between the study populations, since the patient cost of ART differs by country. Although up to three cycles of ART are provided within the Swedish tax-funded healthcare system, women with higher education were more likely to use ART in our study.

In summary, women who have given birth following ART may be at an increased risk of ovarian cancer. However, any risk increase should be considered in relation to the relatively low incidence of ovarian cancer in the general population. Of 1000 Swedish women, five are expected to be diagnosed with ovarian cancer before 65 years of age [28]. Based on the results of our study, seven out of 1000 women with infertility-related diagnoses and non-ART births, and 11 out of 1000 women with ART births are likely to get ovarian cancer before age 65. In other words, the difference in risk between infertile women conceiving with and without ART is only four in a thousand. Which types of infertility, as well as what aspects of the fertility treatment, may predispose women to an increased risk of ovarian cancer after ART should be investigated further.

Women who have given birth following ART may be at an increased risk of ovarian cancer and BOT. Part of that risk seems to be explained by the underlying infertility. Furthermore, since the causes and severity of infertility likely differ between women who go through ART and infertile women who conceive without ART, we are unable to conclude whether the treatment itself could be contributing to the higher risk seen in the women. As ART is relatively new and ovarian cancer most often presents in women of older ages, large population-based studies with infertile comparison groups and longer follow-up are needed in order to confirm or refute our findings.

## Electronic supplementary material

Below is the link to the electronic supplementary material.

Supplementary material 1 (DOCX 44 kb)
