# Supplementary material for: Assisted reproductive technology and risk of ovarian cancer and borderline tumors in parous women: a population-based cohort study
Source: Eur J Epidemiol. 2019 Aug 3;34(11):1093–101. doi: 10.1007/s10654-019-00540-3 (PMC6861355; doi:10.1007/s10654-019-00540-3)
Supplement: Supplementary file 1 — Supplementary material 1 (DOCX 44 kb) [file 10654_2019_540_MOESM1_ESM.docx]

**Supplementary table 1.** Characteristics of the subpopulation with no BOT before follow-up start, by exposure to ART births and infertility

| **Characteristic** | **ART birth 38,003 (2.8%)** | **Infertility,**  **no ART birth 49,183 (3.7%)** | **No infertility,**  **no ART birth 1,252,728 (93.5%)** |
| --- | --- | --- | --- |
| Birth year |  |  |  |
| <1960 | 2,811 (7.4%) | 9,471 (19.3%) | 172,580 (13.8%) |
| 1960-1969 | 12,897 (33.9%) | 14,032 (28.5%) | 454,288 (36.3%) |
| 1970-1979 | 18,709 (49.2%) | 19,088 (38.8%) | 412,417 (32.9%) |
| ≥1980 | 3,586 (9.4%) | 6,592 (13.4%) | 213,443 (17.0%) |
| Infertility diagnosis | 26,460 (69.6%) | 49,183 (100%) | 0 (0.0%) |
| Age at infertility diagnosis, mean (±SD) | 31.2 ± 4.2 | 30.8 ± 5.1 | No diagnosis |
| ART/infertility diagnosis before first birth | 32,442 (85.4%) | 32,533 (66.1%) | No ART/diagnosis |
| Highest education achieved |  |  |  |
| Compulsory school | 1,763 (4.6%) | 4,554 (9.3%) | 107,908 (8.6%) |
| Secondary school | 14,183 (37.3%) | 21,634 (44.0%) | 566,320 (45.2%) |
| Higher education | 21,941 (57.7%) | 22,711 (46.2%) | 554,728 (44.3%) |
| Missing | 116 (0.3%) | 284 (0.6%) | 23,772 (1.9%) |
| Country of birth |  |  |  |
| Nordic country | 33,144 (87.2%) | 41,826 (85.0%) | 1,107,176 (88.4%) |
| Non-nordic country | 4,859 (12.8%) | 7,357 (15.0%) | 145,476 (11.6%) |
| Missing | 0 (0.0%) | 0 (0.0%) | 76 (0.0%) |
| Family history of breast or ovarian cancer | 2,367 (6.2%) | 2,613 (5.3%) | 69,197 (5.5%) |
| Parity at end of follow-up |  |  |  |
| One child | 14,720 (38.7%) | 19,296 (39.2%) | 299,211 (23.9%) |
| Two children | 17,557 (46.2%) | 21,437 (43.6%) | 641,114 (51.2%) |
| Three children | 5,726 (15.1%) | 8,450 (17.2%) | 312,403 (24.9%) |
| Age at first birth |  |  |  |
| <25 years | 2,224 (5.9%) | 8,931 (18.2%) | 419,517 (33.5%) |
| 25-29 years | 7,493 (19.7%) | 14,842 (30.2%) | 475,644 (38.0%) |
| 30-34 years | 15,588 (41.0%) | 15,631 (31.8%) | 271,740 (21.7%) |
| ≥35 years | 12,698 (33.4%) | 9,779 (19.9%) | 85,827 (6.9%) |
| Bilateral oophorectomy | 213 (0.6%) | 347 (0.7%) | 3,623 (0.3%) |
| Salpingectomy | 3,086 (8.1%) | 2,652 (5.4%) | 20,881 (1.7%) |
| Hysterectomy | 545 (1.2%) | 1,137 (2.7%) | 20,843 (1.7%) |
| BMI before index pregnancy |  |  |  |
| <18.5 kg/m^2^ | 678 (1.8%) | 1,430 (2.9%) | 41,880 (3.3%) |
| 18.5-24.9 kg/m^2^ | 20,862 (54.9%) | 24,583 (50.0%) | 652,474 (52.1%) |
| 25.0-29.9 kg/m^2^ | 7,688 (20.2%) | 8,333 (16.9%) | 170,283 (13.6%) |
| ≥30.0 kg/m^2^ | 2,779 (7.3%) | 4,043 (8.2%) | 61,062 (4.9%) |
| Missing | 5,996 (15.8%) | 10,794 (21.9%) | 327,029 (26.1%) |
| Smoking during index pregnancy |  |  |  |
| No | 33,025 (86.9%) | 38,063 (77.4%) | 934,735 (74.6%) |
| Yes | 2,061 (5.4%) | 6,679 (13.6%) | 187,363 (15.0%) |
| Missing | 2,917 (7.7%) | 4,441 (9.0%) | 130,630 (10.4%) |
| Still birth before index pregnancy |  |  |  |
| No | 37,170 (97.8%) | 47,340 (96.3%) | 1,200,289 (95.8%) |
| Yes | 217 (0.6%) | 356 (0.7%) | 4,955 (0.4%) |
| Missing | 616 (1.6%) | 1,487 (3.0%) | 47,484 (3.8%) |

Data presented as number (%) unless otherwise specified. ART: Assisted Reproductive Technology; BMI: body mass index; BOT: borderline ovarian tumor.

**Supplementary table 2.** Associations between infertility diagnosis and ART before and after first birth, and ovarian cancer incidence

| **Population reference** | **Cancer cases** | **Person-years** | **Age-adjusted** | **Multivariable^a^** | |  |
| --- | --- | --- | --- | --- | --- | --- |
|  |  |  | **HR (95% CI)** | | **HR (95% CI)** | |
| No infertility | 894 | 18,211,037 | 1.00 (reference) | | 1.00 (reference) | |
| Primary infertility (no ART) | 42 | 395,382 | 1.53 (1.12-2.09) | | 1.46 (1.07-2.01) | |
| Secondary infertility (no ART) | 14 | 274,953 | 1.28 (0.76-2.17) | | 1.13 (0.67-1.92) | |
| ART for first birth | 35 | 285,214 | 2.19 (1.56-3.07) | | 2.45 (1.71-3.51) | |
| ART for subsequent birth only | 4 | 35,028 | 1.98 (0.74-5.29) | | 2.38 (0.89-6.36) | |

ART: Assisted Reproductive Technology; CI: confidence interval.

^a^Adjusted for age, calendar time, parity, age at first birth, education level, country of birth, family history of breast or ovarian cancer, salpingectomy and hysterectomy.

Likelihood-ratio test for interaction with primary/secondary infertility: Age-adjusted p=0.8273, multivariable p=0.7006.

**Supplementary table 3.** Associations between infertility, ART and incidence of ovarian cancer by subtype

|  | **Cancer cases** | **Age-adjusted** | **Multivariable^a^** |
| --- | --- | --- | --- |
| **Subtype** |  | **HR (95% CI)** | **HR (95% CI)** |
| **Serous** |  |  |  |
| No infertility | 343 | 1.00 (reference) | 1.00 (reference) |
| Infertility (no ART) | 27 | 1.74 (1.17-2.58) | 1.66 (1.11-2.47) |
| ART birth | 14 | 1.91 (1.12-3.26) | 2.08 (1.18-3.65) |
| **Mucinous** |  |  |  |
| No infertility | 133 | 1.00 (reference) | 1.00 (reference) |
| Infertility (no ART) | 7 | 1.32 (0.62-2.83) | 1.10 (0.51-2.40) |
| ART birth | 4 | 1.29 (0.48-3.49) | 1.35 (0.47-3.85) |
| **Endometrioid** |  |  |  |
| No infertility | 85 | 1.00 (reference) | 1.00 (reference) |
| Infertility (no ART) | 4 | 1.09 (0.40-2.97) | 0.94 (0.34-2.58) |
| ART birth | 6 | 3.08 (1.34-7.06) | 3.50 (1.42-8.58) |
| **Clear-cell** |  |  |  |
| No infertility | 34 | 1.00 (reference) | 1.00 (reference) |
| Infertility (no ART) | 4 | 2.52 (0.89-7.14) | 2.97 (1.03-8.59) |
| ART birth | 3 | 3.99 (1.22-13.05) | 8.04 (2.20-29.35) |
| **Other or unspecified carcinoma** |  |  |  |
| No infertility | 87 | 1.00 (reference) | 1.00 (reference) |
| Infertility (no ART) | 6 | 1.52 (0.67-3.49) | 1.27 (0.55-2.94) |
| ART birth | 7 | 3.79 (1.75-8.21) | 3.98 (1.71-9.28) |
| **Non-epithelial** |  |  |  |
| No infertility | 140 | 1.00 (reference) | 1.00 (reference) |
| Infertility (no ART) | 5 | 0.91 (0.37-2.22) | 0.90 (0.36-2.20) |
| ART birth | 5 | 1.73 (0.71-4.24) | 1.95 (0.77-4.96) |

Analyses based on 15,566,155 person-years in the reference group, 583,233 person-years in women with infertility and 316,811 person-years in women with ART birth.

ART: Assisted Reproductive Technology; CI: confidence interval; HR: hazard ratio.

^a^Adjusted for age, calendar time, parity, age at first birth, education level, country of birth, family history of breast or ovarian cancer, salpingectomy, and hysterectomy.

**Supplementary table 4.** Associations between infertility, ART and ovarian cancer incidence, excluding first year of follow-up

| **Population reference** | **Cancer cases** | **Person-years** | **Age-adjusted** | **Multivariable^a^** | |  |
| --- | --- | --- | --- | --- | --- | --- |
|  |  |  | **HR (95% CI)** | | **HR (95% CI)** | |
| No infertility | 867 | 16,983,382 | 1.00 (reference) | | 1.00 (reference) | |
| Infertility (no ART) | 54 | 618,520 | 1.46 (1.11-1.92) | | 1.36 (1.03-1.80) | |
| ART birth | 37 | 288,045 | 2.17 (1.56-3.01) | | 2.46 (1.73-3.49) | |
| **Infertile reference** |  |  |  | |  | |
| No infertility | 867 | 16,983,382 | 0.69 (0.52-0.90) | | 0.74 (0.56-0.97) | |
| Infertility (no ART) | 54 | 618,520 | 1.00 (reference) | | 1.00 (reference) | |
| ART birth | 37 | 288,045 | 1.49 (0.98-2.26) | | 1.81 (1.18-2.78) | |

ART: Assisted Reproductive Technology; CI: confidence interval.

^a^Adjusted for age, calendar time, parity, age at first birth, education level, country of birth, family history of breast or ovarian cancer, salpingectomy and hysterectomy.

**Supplementary table 5.** Associations between infertility, ART and ovarian cancer incidence, among women with information on BMI before index pregnancy

| **Population reference** | **Cancer cases** | **Person-years** | **Age-adjusted** | **Multivariable^a^** | **Multivariable^a,b^** |
| --- | --- | --- | --- | --- | --- |
|  |  |  | **HR (95% CI)** | **HR (95% CI)** | **HR (95% CI)** |
| No infertility | 597 | 12,630,495 | 1.00 (reference) | 1.00 (reference) | 1.00 (reference) |
| Infertility (no ART) | 36 | 481,040 | 1.37 (0.98-1.92) | 1.29 (0.92-1.82) | 1.29 (0.92-1.82) |
| ART birth | 28 | 258,048 | 2.01 (1.37-2.94) | 2.37 (1.58-3.55) | 2.35 (1.57-3.53) |
| **Infertile reference** |  |  |  |  |  |
| No infertility | 597 | 12,630,495 | 0.73 (0.52-1.02) | 0.77 (0.55-1.09) | 0.77 (0.55-1.09) |
| Infertility (no ART) | 36 | 481,040 | 1.00 (reference) | 1.00 (reference) | 1.00 (reference) |
| ART birth | 28 | 258,048 | 1.46 (0.89-2.40) | 1.83 (1.11-3.03) | 1.82 (1.10-3.02) |

ART: Assisted Reproductive Technology; BMI: body mass index; CI: confidence interval.

^a^Adjusted for age, calendar time, parity, age at first birth, education level, country of birth, family history of breast or ovarian cancer, salpingectomy and hysterectomy.

^b^Adjusted for BMI at beginning of first pregnancy resulting in a live birth.

**Supplementary table 6.** Associations between infertility, ART and ovarian cancer incidence, among women with information on smoking during index pregnancy

| **Population reference** | **Cancer cases** | **Person-years** | **Age-adjusted** | **Multivariable^a^** | **Multivariable^a,b^** |
| --- | --- | --- | --- | --- | --- |
|  |  |  | **HR (95% CI)** | **HR (95% CI)** | **HR (95% CI)** |
| No infertility | 773 | 16,405,987 | 1.00 (reference) | 1.00 (reference) | 1.00 (reference) |
| Infertility (no ART) | 49 | 604,832 | 1.47 (1.10-1.96) | 1.36 (1.02-1.83) | 1.36 (1.02-1.83) |
| ART birth | 38 | 295,555 | 2.35 (1.70-3.26) | 2.64 (1.86-3.74) | 2.65 (1.87-3.76) |
| **Infertile reference** |  |  |  |  |  |
| No infertility | 773 | 16,405,987 | 0.68 (0.51-0.91) | 0.73 (0.55-0.98) | 0.73 (0.55-0.98) |
| Infertility (no ART) | 49 | 604,832 | 1.00 (reference) | 1.00 (reference) | 1.00 (reference) |
| ART birth | 38 | 295,555 | 1.61 (1.05-2.46) | 1.94 (1.26-2.99) | 1.95 (1.26-3.00) |

ART: Assisted Reproductive Technology; CI: confidence interval.

^a^Adjusted for age, calendar time, parity, age at first birth, education level, country of birth, family history of breast or ovarian cancer, salpingectomy and hysterectomy.

^b^Adjusted for smoking during first pregnancy resulting in a live birth.

**Supplementary table 7.** Associations between infertility, ART and ovarian cancer incidence in women born in the Nordic countries

| **Population reference** | **Cancer cases** | **Person-years** | **Age-adjusted** | **Multivariable^a^** | |  |
| --- | --- | --- | --- | --- | --- | --- |
|  |  |  | **HR (95% CI)** | | **HR (95% CI)** | |
| No infertility | 834 | 16,822,965 | 1.00 (reference) | | 1.00 (reference) | |
| Infertility (no ART) | 51 | 600,316 | 1.46 (1.10-1.93) | | 1.35 (1.01-1.80) | |
| ART birth | 32 | 287,830 | 1.96 (1.38-2.80) | | 2.25 (1.55-3.27) | |
| **Infertile reference** |  |  |  | |  | |
| No infertility | 834 | 16,822,279 | 0.69 (0.52-0.91) | | 0.74 (0.56-0.99) | |
| Infertility (no ART) | 51 | 599,922 | 1.00 (reference) | | 1.00 (reference) | |
| ART birth | 32 | 287,830 | 1.35 (0.87-2.10) | | 1.66 (1.06-2.61) | |

ART: Assisted Reproductive Technology; CI: confidence interval.

^a^Adjusted for age, calendar time, parity, age at first birth, education level, country of birth, family history of breast or ovarian cancer, salpingectomy and hysterectomy.

**Supplementary table 8.** Associations between infertility diagnosis and ART before and after first birth, and BOT incidence

| **Population reference** | **BOT cases** | **Person-years** | **Age-adjusted** | **Multivariable^a^** | |  |
| --- | --- | --- | --- | --- | --- | --- |
|  |  |  | **HR (95% CI)** | | **HR (95% CI)** | |
| No infertility | 681 | 18,206,107 | 1.00 (reference) | | 1.00 (reference) | |
| Primary infertility (no ART) | 33 | 395,149 | 1.83 (1.29-2.61) | | 1.73 (1.21-2.47) | |
| Secondary infertility (no ART) | 6 | 274,979 | 0.65 (0.29-1.46) | | 0.55 (0.25-1.23) | |
| ART for first birth | 27 | 284,979 | 2.22 (1.51-3.26) | | 2.17 (1.44-3.26) | |
| ART for subsequent birth only | 0 | 34,999 | - | | - | |

ART: Assisted Reproductive Technology; CI: confidence interval.

^a^Adjusted for age, calendar time, parity, age at first birth, education level, country of birth, family history of breast or ovarian cancer, salpingectomy and hysterectomy.

Likelihood-ratio test for interaction with primary/secondary infertility: Age-adjusted p=0.0003, multivariable p=0.0010.

**Supplementary table 9.** Associations between infertility, ART and BOT incidence, excluding first year of follow-up

| **Population reference** | **BOT cases** |  | **Age-adjusted** | **Multivariable^a^** | |  |
| --- | --- | --- | --- | --- | --- | --- |
|  |  | **Person-years** | **HR (95% CI)** | | **HR (95% CI)** | |
| No infertility | 648 | 16,978,606 | 1.00 (reference) | | 1.00 (reference) | |
| Infertility (no ART) | 38 | 618,124 | 1.48 (1.07-2.05) | | 1.34 (0.96-1.86) | |
| ART birth | 26 | 287,796 | 2.06 (1.39-3.05) | | 2.03 (1.34-3.07) | |
| **Infertile reference** |  |  |  | |  | |
| No infertility | 648 | 16,978,606 | 0.68 (0.49-0.94) | | 0.75 (0.54-1.04) | |
| Infertility (no ART) | 38 | 618,124 | 1.00 (reference) | | 1.00 (reference) | |
| ART birth | 26 | 287,796 | 1.39 (0.84-2.29) | | 1.52 (0.92-2.53) | |

ART: Assisted Reproductive Technology; BOT: borderline ovarian tumor; CI: confidence interval.

^a^Adjusted for age, calendar time, parity, age at first birth, education level, country of birth, family history of breast or ovarian cancer, salpingectomy and hysterectomy.

**Supplementary table 10.** Associations between infertility, ART and BOT incidence, among women with information on BMI before index pregnancy

| **Population reference** | **BOT cases** | **Person-years** | **Age-adjusted** | **Multivariable^a^** | **Multivariable^a,b^** |
| --- | --- | --- | --- | --- | --- |
|  |  |  | **HR (95% CI)** | **HR (95% CI)** | **HR (95% CI)** |
| No infertility | 459 | 12,627,331 | 1.00 (reference) | 1.00 (reference) | 1.00 (reference) |
| Infertility (no ART) | 29 | 480,701 | 1.54 (1.05-2.24) | 1.36 (0.93-1.99) | 1.34 (0.91-1.96) |
| ART birth | 19 | 257,878 | 1.76 (1.11-2.79) | 1.71 (1.05-2.77) | 1.66 (1.03-2.70) |
| **Infertile reference** |  |  |  |  |  |
| No infertility | 459 | 12,627,331 | 0.65 (0.45-0.95) | 0.74 (0.50-1.08) | 0.75 (0.51-1.09) |
| Infertility (no ART) | 29 | 480,701 | 1.00 (reference) | 1.00 (reference) | 1.00 (reference) |
| ART birth | 19 | 257,878 | 1.15 (0.64-2.05) | 1.26 (0.70-2.26) | 1.24 (0.69-2.24) |

ART: Assisted Reproductive Technology; BMI: body mass index; BOT: borderline ovarian tumor; CI: confidence interval.

^a^Adjusted for age, calendar time, parity, age at first birth, education level, country of birth, family history of breast or ovarian cancer, salpingectomy and hysterectomy.

^b^Adjusted for BMI at start of first pregnancy resulting in a live birth.

**Supplementary table 11.** Associations between infertility, ART and BOT incidence, among women with information on smoking during index pregnancy

| **Population reference** | **BOT cases** | **Person-years** | **Age-adjusted** | **Multivariable^a^** | **Multivariable^a,b^** |
| --- | --- | --- | --- | --- | --- |
|  |  |  | **HR (95% CI)** | **HR (95% CI)** | **HR (95% CI)** |
| No infertility | 616 | 16,401,511 | 1.00 (reference) | 1.00 (reference) | 1.00 (reference) |
| Infertility (no ART) | 33 | 604,492 | 1.34 (0.94-1.90) | 1.21 (0.85-1.73) | 1.21 (0.85-1.73) |
| ART birth | 25 | 295,353 | 1.96 (1.31-2.92) | 1.90 (1.24-2.89) | 1.92 (1.26-2.93) |
| **Infertile reference** |  |  |  |  |  |
| No infertility | 616 | 16,401,511 | 0.75 (0.53-1.06) | 0.83 (0.58-1.18) | 0.83 (0.58-1.18) |
| Infertility (no ART) | 33 | 604,492 | 1.00 (reference) | 1.00 (reference) | 1.00 (reference) |
| ART birth | 25 | 295,353 | 1.46 (0.87-2.45) | 1.57 (0.92-2.66) | 1.59 (0.94-2.69) |

ART: Assisted Reproductive Technology; BOT: borderline ovarian tumor; CI: confidence interval.

^a^Adjusted for age, calendar time, parity, age at first birth, education level, country of birth, family history of breast or ovarian cancer, salpingectomy and hysterectomy.

^b^Adjusted for smoking during first pregnancy resulting in a live birth.

**Supplementary table 12.** Associations between infertility, ART and BOT incidence in women born in the Nordic countries

| **Population reference** | **BOT cases** | **Person-years** | **Age-adjusted** | **Multivariable^a^** | |  |
| --- | --- | --- | --- | --- | --- | --- |
|  |  |  | **HR (95% CI)** | | **HR (95% CI)** | |
| No infertility | 648 | 16,818,279 | 1.00 (reference) | | 1.00 (reference) | |
| Infertility (no ART) | 36 | 599,922 | 1.42 (1.02-1.99) | | 1.26 (0.89-1.77) | |
| ART birth | 26 | 287,573 | 2.04 (1.38-3.02) | | 1.95 (1.29-2.95) | |
| **Infertile reference** |  |  |  | |  | |
| No infertility | 648 | 16,818,279 | 0.70 (0.50-0.98) | | 0.80 (0.57-1.12) | |
| Infertility (no ART) | 36 | 599,922 | 1.00 (reference) | | 1.00 (reference) | |
| ART birth | 26 | 287,573 | 1.43 (0.86-2.37) | | 1.55 (0.93-2.59) | |

ART: Assisted Reproductive Technology; BOT: borderline ovarian tumor; CI: confidence interval.

^a^Adjusted for age, calendar time, parity, age at first birth, education level, country of birth, family history of breast or ovarian cancer, salpingectomy and hysterectomy.
